# Working from Home and COVID-19: The Chances and Risks for Gender Gaps

**DOI:** 10.1007/s10272-020-0938-5

**Published:** 2020-12-01

**Authors:** Melanie Arntz, Sarra Ben Yahmed, Francesco Berlingieri

**Affiliations:** Labour Markets and Human Resources, ZEW Mannheim - Leibniz Centre for European Economic Research, L7, 1, 68161 Mannheim, Germany

## Abstract

As the COVID-19 pandemic causes a record number of people to work from home, this disruptive event will likely have a long-lasting impact on work arrangements. Given existing research on the effects of working from home on hours worked and wages, an increased availability of working from home may provide a chance for women to catch up with their male counterparts. Yet, the need to simultaneously care for children during the COVID-19 lockdown may also revive traditional gender roles, potentially counteracting such gains. We discuss the likely effects of the COVID-19 pandemic on gender gaps in the labour market and at home in light of recent empirical findings and novel statistics on the heterogeneous structure of work arrangements among couples. We construct a novel teleworkability index that differentiates between fully teleworkable, partly teleworkable and on-site jobs and find that in about a third of households the COVID-19 shock is likely to induce shifts in the intra-household allocation of tasks from mothers to fathers.

The COVID-19 pandemic has disrupted the way many people work. It has notably increased the share of people working from home, creating an unexpected shock to the number of people working remotely instead of on-site. In 2018, the share of employees working at least regularly from home was around 12% in Germany, above 30% in the Netherlands, Finland, Iceland, Luxembourg and Denmark, and around or below 5% in Greece, Italy, Bulgaria and Romania (Eurostat, 2020). These numbers have increased dramatically during the COVID-19 lockdown, although differently across demographic groups. Indeed, since the start of the social distancing measures, many firms have made it possible for their employees to work from home in order to slow down the spread of the coronavirus.[1] Working from home (WFH) has long been considered a means of improving work-life balance, especially for parents. However, during the lockdown, many parents have to combine paid work and full-time childcare, which is likely to reduce their productivity and the expected benefits of WFH. Nevertheless, parents, and especially mothers, could benefit from the increased availability of WFH arrangements thanks to changes in technology and firm culture in the post-COVID-19 era (Alon et al., 2020a).

**Melanie Arntz**, ZEW – Leibniz Centre for European Economic Research, Mannheim; and University of Heidelberg, Germany.

We contribute to recent evidence on the unequal effect of the COVID-19 crisis across gender and parenthood status in several ways. First, to evaluate the labour market effects of the crisis, we construct a novel index that estimates working-from-home feasibility at a detailed occupational level using representative data on tasks and working conditions in Germany. We exploit information on i) the share of employees in an occupation who report that WFH is not possible and ii) the types of tasks performed on the job. This new teleworkability index is the first to differentiate between fully teleworkable, partly teleworkable and on-site jobs. We find that about 31% of jobs in Germany could be almost entirely performed from home while a substantial amount of tasks can be performed from home in 12% of jobs. Women, parents and employees with a university degree are more likely to be employed in occupations with teleworkable tasks. There is evidence that, in April 2020,

1 Fadinger and Schymik (2020) show that higher share of teleworkable jobs is associated with fewer infections and fatalities at the regional level.

26% of German workers were entirely working from home and 35% were combining WFH and on-site work (Grabka et al., 2020; Möhring et al., 2020), just slightly below the WFH potential predicted by our index. This represents a dramatic increase in full-time WFH compared to 2018 when less than 4% of employed individuals worked from home only, and about 12% did so frequently.

Second, we show that WFH preferences are higher among women and parents. About 25% of mothers with children under 13 years of age reported in 2018 that they would like to work from home but were not able to. This share was 20% on average and 21% for women without small children. Based on the gap between preferred and actual WFH take-up, we argue that the rise in remote working will last even after the COVID-19 distancing rules are lifted.

Third, we explore the likely consequences of a rise in WFH arrangements for gender gaps at home and in the labour market in the short and medium run. In the short run, if the rise in WFH is combined with school and day care closures, remote working will likely be a challenging experience for parents. For Germany, we estimate that among 30% of couples with young children, the father has gained more flexibility at work than the mother. In these households, the reallocation of tasks at home, such as childcare and housework, may improve mothers' ability to invest in their careers even in the post-COVID-19 period. Among other households, however, the division of care and housekeeping is likely to remain unequal. Traditional gender roles may even be reinforced as women work fewer hours than their partner on average and have more flexibility in working arrangements in about 28% of households. Still, the increased feasibility of WFH in the future, through current technology adoption and changes in mentality, can benefit women with children as WFH take-up allows them to increase their contractual working hours and monthly earnings.

## How many jobs can be done from home?

Recent studies provide estimates of the share of jobs that could be performed from home during the pandemic. For the US, Dingel and Nieman (2020) estimate that slightly more than 30% of jobs may be carried out from home using the O*Net description of tasks performed on the job for each occupation. Using a similar method, Boeri et al. (2020) estimate this share to be above 30% for the UK and Sweden, around 28% for Germany and France, and just below 24% in Italy. To estimate the share of jobs that can be performed remotely, we employ survey data similarly to Cetrulo et al. (2020), but we exploit information on employees' self-assessment and on the task composition of jobs in Germany.

We describe the data and adopted methodology to compute the *teleworkability index*, i.e. the share of workers who can perform their job from home, by occupations. We draw on the BIBB/BAuA 2018 Employment Survey, a representative survey of about 20,000 adult individuals in paid employment in Germany.[2] First, we calculate the share of individuals reporting that their job cannot be done from home for each 3-digit occupation (German Classification of Occupations, KldB 1992). This information is particularly relevant to identify jobs that can be performed on-site only, what we call the *WFH extensive margin*.[3] Working from home is not possible in the manufacturing and construction sectors, particularly low-qualification jobs such as brick layers, in the transport sector (e.g. drivers), or in the health sector (e.g. nurses). Conversely, the lowest share of employees who report that WFH is not possible is mostly found among high-skill jobs in IT, marketing or consultancy sectors.

Second, we make use of detailed information about the tasks performed on the job to calculate the share of tasks that can be done from home at the occupational level. Specifically, we divide 18 tasks into those that can be done from home (teleworkable tasks) and those that cannot (non-teleworkable tasks) as shown in Table 1. For each individual, we first calculate the share of teleworkable tasks, and then aggregate this individual share to the occupational level. This task-based indicator is informative about the intensity of WFH, i.e. about the share of working time that workers can perform from home, the *WFH intensive margin*. Among the occupations with the lowest share of teleworkable tasks there are either jobs in the manufacturing sector that require special machineries, or service jobs with frequent contact to clients, such as waiters. On the contrary, the occupations with the largest share of teleworkable tasks are office jobs with computer use, such as bookkeepers, office clerks, consultants, architects and lawyers. The final teleworkability index combines both the WFH extensive and intensive margin to distinguish jobs that are i) fully teleworkable, ii) partially teleworkable and iii) non-teleworkable. More precisely, an occupation is defined as teleworkable if more than two-thirds of the tasks can be done from home and less than 20% of individuals in the occupation report that WFH is not possible. An occupation is non-teleworkable if more than one-third of individuals report that WFH is not possible in the job or if more than half of the tasks are non-teleworkable. In the other occupations, WFH is feasible for part of the job, but on-site work is also necessary.

2 The survey was conducted by the Federal Institute for Vocational Education and Training (BIBB) in cooperation with the Federal Institute for Occupational Safety and Health (BAuA).

3 This information has been employed by Alipour et al. (2020), who directly use the share of workers reporting WFH is not possible to infer the share of jobs that are teleworkable.

Table 1
**List of tasks by teleworkability**

| Non-teleworkable tasks | Other, potentially teleworkable, tasks |
|---|---|
| Manufacturing, producing goods and commodities | Providing advice and information |
| Measuring, testing, quality control | Advertising, marketing, public relations, PR |
| Monitoring, control of machines, plants, technical processes | Organising, planning and preparing work processes |
| Repairing, renovating | Developing, researching, constructing |
| Transporting, storing, shipping | Gathering information, researching, documenting |
| Entertaining, accommodating, preparing food | Working with computers |
| Nursing, caring, healing | Use of the Internet or email processing |
| Protecting, guarding, monitoring, regulating traffic | Purchasing, procuring, selling |
| Cleaning, waste disposal, recycling | |

Notes: The group of tasks "Training, instructing, teaching, education" is excluded from the list, since it may be partly but not fully teleworkable.

Source: Authors' elaboration.

Table 2
**Potential working arrangement and actual employment status in 2018**

| | Share of... | | | | | | |
|---|---|---|---|---|---|---|---|
| | Employment | Working time at home | Contractually agreed WFH | Women | Parents with children < 13 | University graduates | Public sector |
| Fully teleworkable | 30.9 | 9.0 | 22.8 | 38.2 | 26.2 | 48.7 | 27.5 |
| Partially teleworkable | 12.0 | 13.8 | 15.7 | 36.4 | 24.1 | 55.4 | 42.1 |
| On-site only | 57.1 | 1.2 | 3.2 | 32.4 | 22.4 | 8.4 | 19.7 |

Notes: All numbers are percentages. BiBB/BAuA 2018 Employment Survey with sampling weights. Employees aged 16-65. The three categories in the left column are based on the teleworkability index described above.

Source: Authors' calculation.

Based on the computed teleworkability index, we find that about 31% of jobs in Germany could be performed from home (Table 2). For another 12% of jobs, a substantial amount of tasks can be performed from home. These jobs could allow, for instance, individuals to work from home 1-2 days per week or to divide the work between on-site and home during a standard workday. Still, more than half of jobs need to be completely carried out on-site. For the education sector, for instance, kindergarten teachers belong to the on-site occupations, school teachers to the occupations that are partially teleworkable and teachers in higher education or adult education are fully teleworkable. The resulting distribution of jobs along the teleworkability dimension varies across demographic groups. Women and parents are slightly overrepresented in jobs that can be (almost) fully done at home. Moreover, employees in fully or partially teleworkable jobs are much more likely to hold a university degree and to work in the public sector.

Table 2 also reports the actual share of working time carried out at home by dividing weekly WFH hours by weekly contracted hours. As expected, workers in on-site occupations spend a very low share of their working time at home (1%), while workers in teleworkable jobs work much longer from home. Interestingly, the average share of time in WFH was higher among the jobs allowing some WFH (14%) than among those allowing full WFH (9%). This is because some of the occupations with the highest share of hours at home are only partially teleworkable, such as school teachers, musicians and religious workers. However, the untapped potential of WFH is largest for the group of fully teleworkable jobs, especially standard office jobs (Grunau et al., 2020).

## Long-lasting changes in working practices

While a certain share of jobs might be theoretically suited for WFH given the main tasks that are performed on the job, it is unclear whether employees and employers actually use this opportunity, or whether they continue working on-site, reduce hours or terminate work contracts. To some extent, these choices are influenced by the need to care for one's children. Before the pandemic, less than 4% of people in paid employment always worked from home, while about 11.5% worked from home at least frequently (Table 3 for 2018). Women, and especially mothers of children under the age of 13, were more likely to use WFH arrangements as almost 5% of them always worked from home, and more than 15% did it at least frequently. The use of WFH has increased substantially during the lockdown. Using the SOEP-CoV survey for Germany, Grabka et al. (2020) report that, among employed individuals in 2019, about 35% were working from home fully or as a complement to on-site work in early April 2020. The share of previously employed individuals who were fully working from home in the same period was 26% according to a German online survey (Möhring et al., 2020). Women were more likely to be (temporarily) out of work,

Table 3
**WFH arrangements and preferences for WFH**

| | | WFH | | | | Not WFH | | |
|---|---|---|---|---|---|---|---|---|
| | | Always | Frequently | Sometimes | Rarely | Would WFH | Would not WFH | WFH not possible |
| Without children under 13 | Women | 4.3 | 7.1 | 8.0 | 6.9 | 21.2 | 12.0 | 40.3 |
| | Men | 2.6 | 6.9 | 7.8 | 8.5 | 17.3 | 7.7 | 49.0 |
| With children under 13 | Women | 4.9 | 10.3 | 8.8 | 7.0 | 25.1 | 8.9 | 34.9 |
| | Men | 3.3 | 9.3 | 11.5 | 9.3 | 18.6 | 6.6 | 41.4 |
| Average | | 3.6 | 7.7 | 8.5 | 7.8 | 19.7 | 9.2 | 43.4 |

Notes: All numbers are percentages. BiBB/BAuA 2018 Employment Survey with sampling weights. Employees aged 16-65.

Source: Authors' calculation.

but also more likely to fully work from home compared to men. Parents were also more likely to work from home as almost 30% of them were working exclusively at home early April. This represents a dramatic increase in full-time WFH compared to 2018. For the US, Brynjolfsson et al. (2020) find from an online survey that about 34% of workers have switched to working from home because of the pandemic, while 4.6% have continued to do so. Moreover, von Gaudecker et al. (2020) find that hours worked from home in the Netherlands increased from 12% to 74% of total hours between the pre-COVID-19 period and May 2020. The exposure to WFH has thus been huge.

About 20% of employed people had reported in 2018 that they would like to work from home. As more firms and employees are now exposed to new ways of working, we expect that the availability of and attitudes towards remote working will improve. In 2018, 43% of employees in Germany reported that WFH was not possible in their job (Table 3). Women and parents with young children were less likely to report that it was not possible. This pattern is consistent with the demographics of teleworkable jobs (Table 2) and might reflect that parents had opted for WFH-friendly occupations or firms. In the post-COVID-19 era, it is likely that WFH will gain acceptance and that fewer employees will report WFH as unfeasible because firms have been forced to make necessary adjustments and investments to enable their employees to work from home.

As for preferences, in 2018, women and parents with young children were more likely to express the wish to work from home. These preferences might evolve depending on their experience during the lockdown. Bloom et al. (2015) study the effects of an experiment that randomly allocated call centre employees to the home or the office. At the end of the experiment, employees were given the choice over where to work, and those who were more productive at home switched to WFH. This highlights the benefits of learning and selection effects when being exposed to new ways of working. It is thus likely that people for whom WFH worked well will adopt WFH on a more regular basis even after the lockdown. As daycare centres and schools are closed, households with and without young children are likely to differ in their WFH experience.

## Working from home and childcare during the lockdown

The closure of schools and daycare centres represents a big challenge for parents who not only have to care for their children, but also need to ensure a minimum level of home-schooling. In 2018, about 22% of households in Germany had at least one child (Eurostat, 2020). This proportion was higher in all other EU member states, the highest being recorded in Ireland (39%), Cyprus, Poland, Slovakia and Romania (above 35%). Fuchs-Schündeln et al. (2020) estimate that the closure of schools and childcare centres affect 11% of workers and 8% of all working hours in Germany. These shares are larger in most other European countries. The ability to be productive at home will depend on the employment situation of both partners and on the number and age of the children. Mothers might encounter more difficulties to work effectively from home given that they usually provide the larger share of childcare at home. In 2018, German couples with at least one child below 13 years had a very unequal division of tasks within the household. Even if both parents were in paid employment (full- or part-time), mothers devoted almost three times as many hours on childcare during a weekday as their male counterparts (5.5 hours vs. two hours on average).[4] Moreover, mothers spent more than one additional hour, or almost twice as long as fathers, on housework during a weekday.

4 Data from the 2014 wave of the SOEP for couples with at least one child under 16 years.

Table 4
**List of tasks by teleworkability**

| | | Father's employment status | | | | | Single mother | Total |
|---|---|---|---|---|---|---|---|---|
| | | Essential job | Work on-site only | Some WFH possible | Fully WFH possible | Not in paid work | | |
| Mother's employment status | Essential | 11.25 | 10.12 | 3.29 | 10.36 | 1.68 | 3.42 | 40.12 |
| | On-site | 2.31 | 2.87 | 0.40 | 0.91 | 0.06 | 2.14 | 8.68 |
| | Some WFH | 1.70 | 1.68 | 0.66 | 0.69 | 0.65 | 0.45 | 5.83 |
| | Fully WFH | 4.29 | 5.71 | 1.41 | 8.01 | 1.20 | 2.85 | 23.47 |
| | Not in paid work | 2.84 | 8.23 | 2.22 | 1.76 | 1.49 | 3.73 | 20.27 |
| Single father | | 0.72 | 0.33 | 0.08 | 0.47 | 0.01 | | 1.63 |
| Total | | 23.12 | 28.93 | 8.06 | 22.20 | 5.11 | 12.59 | 100 / 100 |

Notes: All numbers are percentages. The German Socio-economic Panel (SOEP) 2018 gives the employment composition within households. The list of essential jobs provided by the state of Berlin is matched to the individual data using 3-digit occupation and 2-digit industry codes. WFH possibility is defined using the description of tasks and the share of employees reporting that their job cannot be done from home in 3-digit occupations from the BiBB/BAuA 2018 Employment Survey. Bold figures indicate cases where the mother has less work flexibility than the father. We do not report statistics for same sex couples with children as they are very rare. Code available upon request.

Source: Authors' calculation.

Is the division of tasks within the household likely to be affected during the pandemic? The answer depends on whether fathers are more likely than mothers to reduce their working hours and spend more time at home because of the lockdown. In fact, when the father works from home at least once a week but the mother works on-site only, the time spent on childcare is less unequal and housekeeping is balanced between the two partners. In order to get some ideas about the likely impact of the lockdown on the intra-household division of tasks, we document the mix of parents' employment status in Germany as has been done for the US by Alon et al. (2020a).

Table 4 shows that, in households with children under 13 years, mothers are more likely to be out of paid employment (almost 20%) compared to fathers (5%); these figures include workers in sectors that had to close for several weeks because of the lockdown.[5] Hence, for 28% of households the mother has more flexibility and is likely to spend even more time on household tasks. About 24% of parents live together and have a similar employment status. In these households, women are likely to keep providing more time at home than their partner as 85% of mothers work fewer contractual hours than fathers and more than 60% earn lower hourly wages (SOEP, 2018). In fact, a recent survey among German employees suggests that the pandemic tends to revive traditional role models (Kohlrausch and Zucco, 2020). Compared to pre-COVID-19, on average, it is mainly mothers who have reduced working hours and increased the time spent on childcare while corresponding adjustments among fathers turn out to be much smaller.

However, mothers are also more likely to hold an essential job (40%) than fathers (23%), which reverts the traditional role models.[6] In more than 25% of households, the mother works in a sector that has to continue operating while the father does not. In another 4% of households, the mother has less flexibility than the father (on-site vs. WFH; work on-site or at home vs. out of work). Hence, in about 30% of households the COVID-19 shock is likely to induce shifts in the intra-household allocation of tasks from mothers to fathers.[7]

## What will be the medium-term consequences of the increase in remote working?

Given the recent exposure to WFH and preferences for this work arrangement, it is likely that the share of people working remotely remains high even after social distancing rules are alleviated and schools open again. Both theory and empirics suggest that the possibility to work from home increases labour supply at the intensive margin, especially among mothers (see Arntz et al., 2019, for a review and

5 These include shops not essential to secure basic supplies, restaurants and cafés, hotels and pensions, services in body care, sports and leisure facilities, bars and clubs, (trade) fairs, music schools, driving schools, theatres, concert halls and opera houses.

6 For the classification of essential occupations we follow the list published by the state of Berlin as of 22 April 2020.

7 Summing up all cases where the mother has less work flexibility than the father; figures in bold in the table.

new results for Germany). In addition, experiments at the firm level show that WFH may increase worker productivity (Bloom et al., 2015; Angelici and Profeta, 2020). In fact, the need to organise remote work on a grand scale during the coronavirus pandemic is likely to establish new and highly productive forms of remote work that may even substitute for certain on-site activities or business meetings. The costs and benefits of WFH are likely to be re-evaluated in light of the current experiences. In this case, pre-COVID-19 findings on the effects of WFH on hourly wages may change. So far, the evidence shows that WFH comes with a wage premium only if it increases contractual hours, as has been shown for fathers in Arntz et al. (2019). For mothers, until now, similar benefits are restricted to those who simultaneously change employer. Conversely, WFH may even bring a penalty if it is used to do overtime only, despite potential signalling and productivity effects. With new work organisations and a broader adoption of digital technologies, productivity at home is likely to increase. Thus, overtime done from home is more likely to be associated with positive wage compensation in the future.

Even if workers, especially women with young children, value flexible work arrangements (Mas and Pallais, 2017; Angelici and Profeta, 2020), WFH may generate new sources of conflict and stress at home (Song and Gao, 2019). Arntz et al. (2019) find that, in Germany, employees without children under the age of 16 who start WFH do an extra hour per week of unpaid overtime and still report higher job satisfaction. On the other hand, parents with young children increase their contracted working hours and report no significant change in job and life satisfaction.

## Conclusion

Overall, the effects of WFH arrangements depend a lot on the presence of dependent children, and the current situation is likely to exacerbate these differences. For parents, WFH during the lockdown may result in a negative experience with a potential increase in conflicts between work and family needs. However, as childcare facilities and school open again, parents could fully benefit from a higher availability of WFH arrangements as the required technology has been adopted and firm culture has changed.

Based on the findings of Arntz et al. (2019) for Germany, more WFH opportunities could help reduce gender gaps in hours worked and wages in the labour market. These gains might even be larger after the lockdown for two reasons. First, WFH is likely to be seen less as a means to accommodate private needs, and more as a means to organise certain types of tasks efficiently in a way that is beneficial to the firm. Second, the intra-household reallocation of tasks during the lockdown may have long-lasting effects that will benefit women in households where the father has increased the time spent on childcare and housework (Alon et al., 2020b). However, for a substantial share of households, the lockdown may strengthen or even revive traditional gender roles, hence potentially inducing negative effects for women's future career prospects. Digging deeper into the heterogeneous impact of COVID-19 on female and male labour market outcomes will thus be an important path for future research.
